# Vipera Snakebite in Children: A Focus on Europe

**DOI:** 10.3390/children12030393

**Published:** 2025-03-20

**Authors:** Greta Orlandi, Nadia Rossi, Francesco Chiarelli, Paola Di Filippo

**Affiliations:** Department of Pediatrics, University of Chieti, 66100 Chieti, Italy; gretaandrea.orlandi@studenti.unich.it (G.O.); nadia.rossi@asl2abruzzo.it (N.R.); chiarelli@unich.it (F.C.)

**Keywords:** viper bite, snake envenomation, antivenom, antidote, children

## Abstract

Although there are over 5 million cases of snakebites each year, up-to-date data on epidemiology and management in European children are lacking in literature. Snakebite envenoming is a rare but potentially life-threatening event, and children are more susceptible due to their lower weight-to-venom ratio. Symptoms of viper envenomation in children are mainly local, but the lymphatic and blood diffusion of the venom may cause systemic symptoms, mainly hemotoxic and cytotoxic symptoms. Immunotherapy with anti-viper serums is the cornerstone of treatment for viper bites, while the use of antibiotics, steroids and analgesics is still unclear and unstandardized. Recently, efforts have been made to improve the pediatric approach to viper envenomation in European children. Several pediatric case reports in children were reported in literature, and a pediatric grading severity score and electronic clinical tool (VipGrade^®^) were created to better manage this issue. However, larger studies are needed to validate these pediatric tools. This narrative review focuses on the clinical characteristics and management of European snake envenomation in children.

## 1. Introduction

In 2009, the World Health Organization (WHO) added snakebites to the list of neglected diseases, and antisnake venom was included in the list of essential drugs. To date, the available epidemiological data on snakebites are still incomplete and inaccurate [1].

Current and up-to-date articles on snakebites and their treatment in European children are lacking in literature, even though there are over 5 million cases of snakebites each year [2]. The relatively low amount of data about snakebites in Europe likely contributes to a lack of sufficient interest in the issue, also indicated by the absence of mandatory reporting and the standardization of notification by health services [3]. Furthermore, epidemiological data on snakebites are often unreliable, as most bites occur in rural areas where medical facilities are scarce, and many victims seek treatment from traditional healers. As a result, the number of cases indicated in hospital records is lower than the actual number [4]. Snakebite envenoming is a rare but potentially fatal event, killing between 81,000 and 138,000 people each year and leaving another 400,000 people worldwide with permanent disabilities [2]. Globally, most cases are caused by species from the Elapidae and Viperidae families [5].

One-quarter of all viper bites in Europe occur without venom inoculation (dry bites) and clinical consequences, but lead to alarmism in the patient and physicians [6,7].

In the remaining three-quarters of cases, the injection of venom causes predominantly local symptoms. However, systemic symptoms may be present due to the venom’s lymphatic and blood diffusion. Furthermore, symptoms of viper-bite envenomation vary depending on the anatomical site involved, the amount and characteristics of the venom and the general condition of the victim [8].

Snakebite envenoming in children, although proportionally less frequent, is generally more severe and often results in worse outcomes [9]. Children are at higher risk compared to adults due to their smaller body size [8].

In this narrative review, we describe the state of the art regarding snake envenomation in Europe and the related clinical framework and management of viper bites in children. Indeed, recognizing the clinical characteristics of a viper bite has a key role in prompt recognition and adequate treatment with anti-viper serum and supportive care.

## 2. Geographical and Epidemiologic Characteristics of Snakebite

More than 95% of snakebite cases occur in tropical settings, particularly in South Asia, sub-Saharan Africa and South America, with the highest percentages occurring in low-income rural populations and children [10].

In tropical countries, snakebites are considered an occupational hazard for agricultural workers, with the highest incidence and mortality reported in southern (121,000 cases and 14,000 deaths per year) and Southeast Asia (111,000 cases and 790 deaths per year) [11].

In contrast, the USA records around 9000 cases and 5 deaths per year [1].

The incidence of snakebites in Europe is lower than in the rest of the world (0.22–1.43 affected individuals per 100,000 people per year) [7], and lethality is reduced due to the high quality of public health systems in these countries and the lower lethality of native snakes compared to their foreign counterparts [12].

In Italy, the incidence can reach 5 per 100,000, with morbidity around 1 per 100,000 and a mortality rate of 0.1–0.2%. The Italian National Health Institute reports only one case of death due to venomous snakebites between 2003 and 2006 [13], and a more recent Italian case series declared no mortality in children [7].

Venomous snakes are classified into four families: Viperidae, Atractaspididae, Elapidae and Colubridae. Viperidae consist of more than 200 species, among which are some of the most dangerous snakes to human beings, including the Russell’s viper, the carpet viper, the puff adder, rattlesnakes and lance-head pit vipers [14]. Viperidae are distributed over the Americas, Europe, Africa and Asia [14].

The genus Vipera belongs to the “true vipers” and includes more than 20 species, occurring in virtually every country in Europe, except for some large islands such as the Balearics, Corsica, Crete, Malta and Sardinia [6]. Venomous snakes in Europe all belong to the genus Vipera and are all true vipers [3], unlike their American counterparts, which include pit vipers [6,15].

The species *Vipera berus* is the most common European viper, followed by *Vipera ammdytes*, *Vipera aspis* and *Vipera ursinii*, [7,16]. *Vipera* (*V.*) *aspis* is the most common viper in Italy, while *V. ursinii* is most common on the Apennines [7,16]. European vipers have peculiar characteristics useful for their identification, such as an average size of 50−70 cm, a vertical pupil which in bright light assumes a slit shape, at least one row of subocular scales, small scales irregularly arranged covering the top of the head, dorsal keeled scales of the trunk and a triangular or sub-oval head shape [17].

In a recent systematic review, a precise identification of the snake, indicating the genus and species, was reported for 56.8% of snakebites in Europe, and the most involved taxon was *V. berus* (63.3%), followed by *V. ammodytes* (17.7%), *V. aspis* (11.7%), *V. latastei* (4.0%), *V. ursinii* (2.6%) and *V. seoanei* (0.7%) [18]. As snakebites are more prevalent in the summer and during the rainy season in association with agricultural activities, they can be classified as occupational diseases [12].

In Europe, most of the snakebites occurred in June (75.7%) and accidentally, while working, walking or playing in a rural area. In 22.7% of cases, the bite occurred while the victim was catching or feeding a pet snake [18]. Male gender, living in rural areas, summer months and daytime are the main risk factors for viper bites [13]. Although snakebites can occur at any age, an Italian survey showed that they are more common in pre-pubertal children, with an average age of 6.5 years, due to their outdoor activities. Prevalence is lower in preschoolers (0–5 years), and children under 3 are generally spared as they stay at home and are usually accompanied by caregivers when outside [19].

## 3. Pathogenesis and Clinical Characteristics of Snakebite in Children in Europe

Snakes inject their venom subcutaneously or intramuscularly, rarely intravenously [20]. The pathogenesis of Vipera snakebites varies according to different venom compositions. In particular, the main Vipera toxins are as follows:

1. Phospholipase A2, responsible for hemolysis, neurotoxicity, myotoxicity, cardiotoxicity, cytotoxicity, anticoagulation, convulsions, hypotension and inflammation;
2. Snake venom serine proteinases (thrombin-like enzymes) and snake venom metalloproteinases, leading to local and systemic hemorrhages, although some classes of snake venom metalloproteinases may also cause procoagulant and pro-inflammatory activities;
3. Snake C-type lectin-like proteins, with anticoagulant and platelet-modulating activities;
4. Disintegrins, responsible for cell adhesion, migration, apoptosis, platelet aggregation and angiogenesis [3,6,18,21,22,23].

Generally, viperid venoms mainly induce hemotoxic and cytotoxic symptoms, more rarely neurotoxic effects [6], which typically follow Elapidae bites [7]. The hemorrhagic effect is caused by direct interference with the coagulation cascade and the consumption of platelets and coagulation factors at the site of injection [7]. The neurotoxic effect is due to phospholipase A2 neurotoxins, which could be in the venom of *V. ammodytes* and some populations of *V. aspis* [24,25].

The severity of clinical manifestations depends on factors such as the snake size, the number of bites, the nature, location and depth of the bite and the amount of venom injected [1]. The symptoms also depend on the involved viper species because of the different toxicological profiles [26]. Therefore, the identification of the offending snake species may predict the clinical course. The identification of the species in Europe is facilitated by the fact that many areas of Europe are inhabited by only one species of venomous snake, especially in Northern and Central Europe [27]. However, the composition of snake venom can vary within genera and even within species due to ontogeny and geographical distribution, making the clinical evolution of snakebites unpredictable [27].

Although the variability in snake venom composition and lethality within the same species and/or for a given species is known, the lethal dose 50 of all viper snakes usually ranges from 1.0 to 2.0 mg/kg [7]. The venom glands of adult specimens of *V. ammodytes* have 10–45 mg of venom, and one bite can excrete as much as 20 mg of venom, which can be a lethal dose, especially for children and patients with chronically illnesses [28]. Indeed, due to their lower weight-to-venom ratio, children are more susceptible to the potentially fatal effects of snakebites; this can also lead to more fast and severe neurotoxicity, coagulopathy and severe local tissue damage [20]. Furthermore, female gender, intense pain at onset, being bitten in the afternoon, elevated glycemia and an upper-extremity location are associated with high-grade envenomation in children [29].

**Figure 1:**
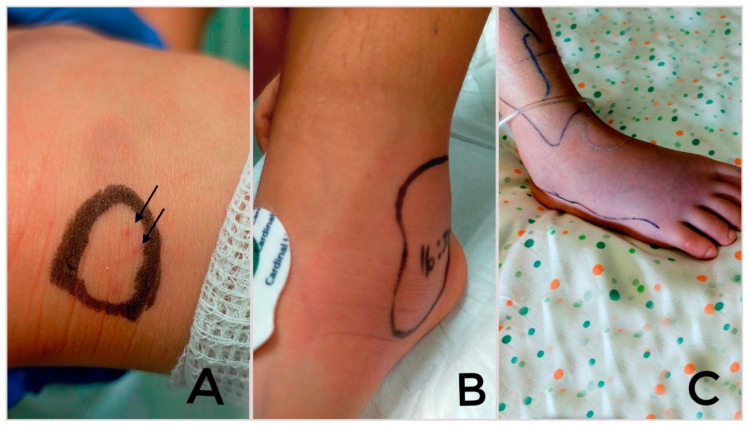
Evolution of the lesion on the right ankle of a child: the viper bite, defined by the imprint of the reptile’s two hooked teeth (**A**), and the local edema on the first day (**B**) and on the fifth day (**C**).

The first skin manifestation of viper envenomation is 1–2 distinct holes caused by the fangs [6] (Figure 1). In a systematic review of 3574 European studies (including 28 studies of children and teenagers), evidence of fang marks was present in 90.5% of cases; 98.3% of snakebites involved the limbs, and the upper limbs were the most involved anatomic site (53.1%) [18]. Venom cytotoxicity induces the disruption of blood vessel integrity, causing the appearance of local signs after 30 min, including edema (73.6% of cases), ecchymosis (68.4%) and erythema (56.6%) [7,18]. Loco-regional adenopathy was reported in 42.7% of cases [18]. Skin and muscular necrosis, hives, purpura, petechiae and acute compartment syndrome may appear in later stages [6,7,20].

Compartment syndrome occurs rarely but can cause ischemic damage to nerves and muscles, with irreversible tissue loss if not treated with a prompt fasciotomy [8]. However, the onset of compartment syndrome may take several hours or even days to develop after snakebite, requiring careful monitoring over time. The lack of reliable predictors for compartment syndrome makes management challenging and costly. In a retrospective study, Hsu CP et al. [30] identified elevated white blood cell counts and aspartate aminotransferase levels as risk factors for the development of compartment syndrome in 136 snakebitten adults.

Although rare, lymphatic and blood toxicity can lead to systemic complications which can be life-threatening [13]. Fever, headache, skin rash, vomiting and diarrhea are the most common systemic symptoms, but coagulopathy, bleeding, hypovolemia, acute kidney injury, cranial nerve neurotoxicity, dysesthesia/paresthesia and cardiac ischemia can also be observed [6,20]. In severe cases, hypovolemia secondary to coagulopathy, capillary leakage, and vasoactive and myocardial-depressing toxins may cause cardiovascular shock [20].

Although neurological consequences following viper envenomation are uncommon in children, the involvement of the III, IV and VI cranial nerves may cause ptosis, diplopia and chewing disorder [24]. Other neurological symptoms such as segmental dysesthesia or paresthesia occurred rarely [18]. Neurological symptoms appear at least 11 h after the bite even in adults with mild local symptoms, while in children, neurotoxicity is always associated with extensive local effects [31].

A rare case of spherocytosis with severe intravascular hemolysis, hemoglobinemia and hemoglobinuria after a *V. ammodytes* viper bite in an adult farmer was recently reported in Greece. The observed laboratory changes (rapid fall in hematocrit, along with hyperbilirubinemia, hemoglobinuria, absent haptoglobin and a marked increase in lactic dehydrogenase) were consistent with intravascular hemolysis, but the hematological picture was consistent with spherocytic rather than microangiopathic hemolysis [32]. To date, no similar cases have been described in children.

A grading system for assessing the severity of viper envenoming can be used as a guideline in research and in certain clinical settings. Based on the severity of viper bites, Audebert et al. [33] developed a scale, which Boels et al. [34,35] later modified, identifying four grades:

- Grade 0 (no envenoming): traces of bite and local signs.
- Grade 1 (mild envenoming): local edema and absence of systemic symptoms.
- Grade 2 (moderate envenoming): extensive regional edema, hypotension, vomiting, diarrhea.
- Grade 3 (severe envenoming): persistent hypotension or shock, hemorrhage.

In 2021, Marano et al. [7] proposed a grading severity score adjusted for pediatric patients (pGSS) with relative management indications, which could be a useful tool for emergency pediatricians.

In the aforementioned systematic review, grade 2 was the most frequently reported grade of envenomation (42.2%), followed by grade 1 (32.3%), grade 3 (13.9%) and grade 0 (11.7%) [18].

## 4. Long-Term Consequences of Snakebite in Children

Most snakebite patients are not followed up with after they have been discharged from the hospital and the acute effects have resolved. Nevertheless, snakebites can also lead to long-term consequences, defined as conditions that persist or manifest more than six weeks after envenomation [36].

Waiddanatha et al. [36], in their systematic review of 51 studies conducted on adults and children in different countries around the world, described the long-term effects of snake envenomation. These effects include disabilities resulting from amputations, deformities, contractures and chronic ulceration, with malignant changes being rare. However, such complications are mostly related to bites from African and Asian cobras, and Central and South American pit vipers [36]. Similarly, bites from Russell’s viper in Sri Lanka, India and Myanmar were associated with the progression of acute kidney injury to chronic renal failure, as well as endocrine issues like delayed hypopituitarism [36,37].

Long-term psychological effects (depression, post-traumatic stress disorder and somatization), as well as long-term neurological complications like ptosis, were also described in children [38], but these too are not associated with European viper bites.

To date, no long-term sequelae have been reported in literature after European viper bites in children [7], even after an acute phase characterized by compartment syndrome [8].

## 5. Diagnosis of Snakebite Envenomation

The diagnosis of snakebite envenomation includes a thorough patient history, a targeted examination and appropriate laboratory testing.

A detailed history includes information about the circumstances of the bite (geography, time of incident, activity and number of bites), details about the snake (if seen, carried or photographed), clinical manifestations of envenomation (including time of onset), first aid administered and past medical history (allergies, previous snakebites, pertinent medications and pre-existing medical conditions) [27].

Hematologic changes are consistently observed after viper bites, likely as a response to the toxins or acute stress [13]. Laboratory testing includes a blood coagulation profile and complete blood count to screen for venom-induced coagulopathies and anemia induced by internal bleeding. Other laboratory tests are useful in identifying complications, such as creatine kinase, electrolyte, urea and creatinine/nitrogen levels, which, together with urinalysis, can be used to evaluate venom-induced rhabdomyolysis and associated complications, such as myoglobinuric renal failure or polyuria, oliguria or anuria [27].

In case of a viper bite, the recommended blood tests every 6 h for 24 h include those for hemocoagulation, blood count, urinalysis, hepatic tests, renal function, electrolytes, lactate dehydrogenase and creatine phosphokinase. Furthermore, laboratory tests help to assess the severity of envenoming and, thereby, the need for antivenom [6].

In literature, leukocytosis associated with increased neutrophils is associated with the clinical severity of viper envenomation in children [7]. It is also the major hematological change in a case report of a child with acute compartment syndrome needing fasciotomy after a bite by *V. walser* in the north of Italy [8].

A study by Ozay et al. [30] involving 77 children bitten by venomous snakes in Turkey found that 21% had thrombocytopenia (<120,000/mm^3^), and 54% had leukocytosis. Additionally, the study revealed elevated levels of aspartate aminotransferase, lactate dehydrogenase and creatine phosphokinase and an increased prothrombin time, while the mean activated partial thromboplastin time was lower than the normal range. The platelet count was found to be inversely related to the severity of the grading score and the length of hospitalization [39].

Digital oximetry and an electrocardiogram are also recommended [6].

## 6. Pediatric Management of Snakebite

### 6.1. Pre-Hospital Care

In a pre-hospital setting, the rapid transport of the child to the nearest medical facility is the priority. It is important to keep the child calm and comfortable, as a hyperdynamic state can accelerate venom spread [40]. Practices such as incision, suction devices, snake stones, cryotherapy and tourniquets should be avoided [20] as they are not supported by scientific evidence and can delay medical attention, alter the clinical presentation and increase the risk of complications [3]. Tourniquets are often still applied in rural settings, but they can increase local tissue destruction and cause gangrene [20]. Pressure immobilization bandages are useful for elapid bites (which do not cause local swelling) to reduce lymph flow, but are not recommended for viperid bites because excessive pressure can sometimes worsen local tissue damage. Therefore, the WHO does not recommend pressure bandages for most snakebites, although limb immobilization plays a key role in treatment for all snakebites [20]. The affected limb should be immobilized in a functional position, ideally below the level of the heart, to reduce the lymphatic absorption of the venom [40].

Therefore, in a pre-hospital setting, the only beneficial actions include cleansing the wound with water and alcohol-free detergents, removing any constricting objects (e.g., jewelery, watches) that might impede blood circulation in case of swelling and applying a mild immobilization bandage, applied solely by emergency personnel [3].

Immediate medical attention should be sought to ensure airway patency and provide adequate respiratory support, while preventing the aspiration of vomit or fluids [40].

### 6.2. Hospital Care

Upon arrival at the hospital, an ABCDE approach should be followed. A detailed history should be taken, including the time of the incident and a description of the snake. First aid measures should be reviewed, and the patient’s general medical history, including any food or drug allergies, should be assessed [20].

Minimal or no pain without additional symptoms suggests a dry bite; in this case, a 6 h observation period may be recommended [12]. Significant neurologic impairment could require prompt intubation and ventilation [12].

### 6.3. Immunotherapy

The WHO and the *South East Asian Region Organization* guidelines defined treatment for viper bites [12].

Immunotherapy, which includes anti-viper serums, is the cornerstone of treatment for viper bites and is prescribed in case of at least grade 1 envenomation [6]. Antivenoms are preparations of immunoglobulins or immunoglobulin fragments arranged by immunizing large animals (horses or sheep) with snake venom. After blood collection and plasma separation, antibodies are purified and preparations formulated to have a standard neutralizing potency against venoms used in immunization [41]. Available antisera are generally manufactured by local facilities that create antivenoms specific for the viper species populating the area [26].

Indications for administering antivenom (equine Fab or ovine Fab) include the following: circulatory instability unresponsive to treatment, rapid progression of swelling or bruising, prolonged or recurrent gastrointestinal symptoms, mucosal swelling, fluctuating consciousness and nerve paralysis (peripheral or cranial) [13]. In borderline cases, the following conditions may further support the need for antivenom: leukocytosis (>15–20 × 10⁹/L), metabolic acidosis, hemolysis, coagulation issues and electrocardiographic abnormalities [13].

The anti-viper serum might be administered within four hours after the bite but is still effective if taken within twenty-four hours [12].

Identifying the snake species would be desirable in order to choose the most suitable antivenom. When the offending snake species is known, monovalent antivenoms may be administered, while polyvalent antivenoms are useful in cases of unidentified snake species. However, the relative proportion of antibodies to the target toxin in a polyvalent antivenom is often not as high as in a monovalent antivenom for a given snake species, resulting in reduced efficacy. Therefore, a higher dose of a polyvalent antidote may be required than of a monovalent one, resulting in increased costs and the risk of developing adverse reactions. Unfortunately, polyvalent antivenoms are often preferred, both because of the lack of monovalent antivenoms and because of the difficulty in identifying with certainty the offending snake [27].

When a polyvalent antivenom is not available, monovalent antivenoms may be administered, exploiting their cross-reactivity [42]. Indeed, antivenom raised against venom from one species can be effective against venoms from other vipers indigenous to Europe [27], because most antivenom serums can neutralize the venoms of multiple snake species [7].

The antivenom derived from the venom of *V. ammodytes*, *V. berus* and *V. aspis* seems to be the best option for treating viper bites in Italy as well as in Europe, where these species of vipers occur [42].

The composition of snake venom and the protein sequences of homologous toxins are generally characterized by considerable diversity [43]. Recent genetic and proteomic analyses showed that PLA2 components were found in both *V. aspis* and *V. berus* venom, but only those contained in *V. aspis* venom are neurotoxic [31]. Therefore, PLA2 present in venoms of different species could be functionally and antigenically different and therefore variably susceptible to neutralization by available antisera. A recent Italian study investigated the toxicological profile of *V. aspis* and *V. berus* venoms and the effectiveness of two monovalent antivenoms in vivo in mice. The two antivenoms tested effectively counteract the effect of *V. berus* venom, but only partially neutralize the neurotoxicity of *V. aspis*, supporting the use of polyvalent antivenoms [26].

Therefore, the use of polyvalent antivenoms against numerous species and subspecies of vipers could represent an interesting strategy for the more effective management of European snakes. Furthermore, small-molecule inhibitors that target the activity of the main toxic components of venoms can also be taken into account for the development of new broad-spectrum antivenoms [26].

The antidote should ideally be administered via intravenous infusion; if this is not possible, it can be administered by slow intravenous push at a maximum rate of 2 mL/minute. The doses of antivenom in children and adults are the same, since the volume of venom injected does not depend on the size of the victim. However, the volume of saline in which the antivenom is diluted is generally lower in children to avoid fluid overload [20].

An additional dose of antivenom can be administered if neurotoxicity or cardiocirculatory symptoms worsen or if bleeding persists 1–2 h following the initial dose [42].

However, a recent systematic review of articles relating to antivenom in Europe showed that no randomized control trials comparing the effectiveness of antivenoms were carried out and clinical studies were mostly retrospective. Better and more systematic data, including randomized controlled trials comparing different antivenoms in Europe, are required to better assess the antivenoms’ effectiveness against different Vipera species [44].

### 6.4. Adverse Reactions to Antivenom

Potential serious life-threatening reactions due to the use of foreign animal proteins derived from the immunized animal are the main issue with antidotes [45].

Adverse reactions to antivenom can be classified into early and late reactions [46].

Early reactions tend to be mild and include symptoms such as itching, nausea, vomiting, diarrhea, headache or fever [43]. However, in some cases, severe systemic anaphylaxis can occur, with bronchospasm, hypotension or angioedema [45]. In one study, anaphylaxis occurred in 3.3% patients within 10–180 min after the administration of antivenom [18].

These early reactions are likely caused by complement activation through immunoglobulin(Ig)-G aggregates present in equine serum [47]. Modifying antivenoms through enzymatic digestion with papain or pepsin (which cleaves IgG) and the subsequent removal of the Fc region may reduce, but not completely eliminate, acute reactions [48].

In cases of early reactions, subcutaneous adrenaline administration can be helpful. The incidence of early reactions varies, ranging from 2% to 50% of patients [45].

Late reactions, similar to serum sickness, can occur 1–2 weeks after treatment. These are characterized by fever, itching, arthralgia, lymphadenopathy and albuminuria. They are caused by immune complexes and typically respond to treatment with corticosteroids and antihistamines [45].

However, in literature, these reactions are mostly described after the administration of antivenom outside Europe. Data from a European systematic review showed that adverse reactions were reported in 37 of 2408 cases (1.5%), including 7 cases of anaphylaxis. Therefore, reported adverse reactions were rare, suggesting that modern intravenous antivenoms are of good quality [44].

### 6.5. Ancillary Therapy

In addition to immunotherapy, ancillary measures and drugs may play a role in the management of viper bites.

The involved skin area can be cleaned with hydrogen peroxide, potassium permanganate or plain water (viper venom is water-soluble) [6]. Alcohol and other chemical substances should be avoided since toxic compounds can be generated [3]. The use of tourniquets, cutting, sucking or scarifying the wound and the application of chemicals or electric shock are not recommended [6]. It is essential to periodically evaluate vesicles and blisters, because they can hide underlying necrosis [6].

Antibiotic usage is controversial and should be evaluated based on the patient’s medical history, physical examination and laboratory results [1]. A swab of the affected area is recommended for diagnostic purposes [3]. Corticosteroids should not be administered [20] because they increase the risk of bacterial infections [3].

Tetanus toxin and tetanus immunoglobulin should be administered to unvaccinated children. Ensuring adequate pain relief is crucial, even though some snake venoms cause little or no pain. In low- and middle-income countries, ketamine is commonly used for analgesia [1], but paracetamol is preferable for analgesia in children [20]. Nonsteroidal Anti-Inflammatory Drugs should be avoided due to their potential to increase bleeding risk [3].

Despite the recommendations, a recent systematic review found that antibiotics (in 2009/3065 cases), steroids (in 1385/2480 cases) and antihistamines (in 913/1737 cases) were frequently administered. Notably, systemic steroids were frequently co-administered with antivenom. Many patients received fluids and analgesics, as well as heparin (139/1287 cases) and inotrope drugs (82/732 cases) [18].

The use of anticoagulants in the treatment of viper bites remains controversial. Some studies suggest that low-molecular-weight heparin (LMWH) may increase the risk of hematoma and functional impairment, particularly when patients have received early envenomation treatment [49]. A recent study by Boels et al. [34] found that prophylactic administration of LMWH was significantly associated with longer hospital stays, exceeding 48 h.

## 7. Pediatric Viper-Bite Cases in Europe in Literature

Most pediatric case series of snakebites have been conducted in India, Sri Lanka and other non-European countries, where this issue is more frequent. Snakes in Europe all belong to the genus true Vipera and lead to different clinical features compared to snakes such as pit vipers [3,15]. Information about snake envenomation in Europe is mainly derived from studies in adults. Recently, several case series and studies regarding European viper envenomation in children were reported in literature [7,8,29,50,51]. These data helped in understanding the specific characteristics of snake venom envenomation in children in Europe and in developing specific tools, such as the pediatric grading severity score [7].

Marano et al. [7] retrospectively reviewed 24 cases of pediatric viper bites admitted to the Pediatric Emergency Department and the Pediatric Intensive Care Unit of the Bambino Gesù Children Hospital in Rome between 2000 and 2020. The median age of the patients was 4.2 years, with a male predominance (female-to-male ratio of 1:1.6), and most cases occurred in the late summer. All extremities were involved, with a slight predominance of the superior limbs (54.5%). The most frequent signs were pain, local edema and swelling, and no mortality was observed. Two patients presented neurological signs (ptosis and dysarthria or nystagmus). The authors also observed that a higher white blood cell count and an increase in the International Normalized Ratio, lactate dehydrogenase and C-reactive protein levels were associated with clinical severity. Antivenom administration was necessary in 12 cases, and 4 patients required a second dose; no significant side effects were reported, except in 1 case of serum sickness with a mild rash on both legs a week after the administration of the antivenom.

Recently, the case of a 6-year-old Italian girl who experienced multiple viper bites in the lower limb and developed compartment syndrome was described. This case showed that a prompt assessment of the severity of viper envenomation and the administration of antivenom are essential in children [8].

A French retrospective study, including 58 children (43 males) who arrived at the emergency department between 2001 and 2009 with a mean age of 8 years, documented that bites occurred mainly in the afternoon (62%) and mainly affected the lower limbs (77%). The degrees of envenomation were grades 0 and 1 in 83% of cases and grades 2 and 3 in 17% of cases [29]. A potential association between initial hyperglycemia, being bitten on an upper extremity, being bitten during the afternoon and experiencing severe pain and the risk of progression to high-grade envenomation was found [29] and confirmed in another retrospective study conducted between 2001 and 2014 with 83 children [50].

Interestingly, a French electronic clinical tool (VipGrade^®^) useful to grade envenomations due to European Vipera and decide whether to use specific immunotherapy was created in 2022. However, a retrospective cohort of 116 children with Vipera envenomation showed a discordance of this tool with the initial grading assigned by clinicians on admission, notably in grade I, overgrading a substantial number of cases [52].

A larger European pediatric cohort was investigated in a retrospective study of 160 children that experienced venomous snakebites in southern Croatia [52]. The authors confirmed that most bites occurred from early May to late August and the greater involvement of upper limbs. Local complications were observed in 24% of children and included hemorrhagic blisters (20%) and compartment syndrome (7.5%). General complications were observed in 25% of patients and included cranial nerve paresis or paralysis (11.2%) and shock symptoms (7%). Only one child (0.6%) died because of snakebite directly on the neck. All patients received antivenom without an anaphylactic reaction. All patients also received tetanus prophylaxis, and almost all of them received antibiotics, corticosteroids and antihistamines [51].

## 8. Conclusions

Being an uncommon event in Europe, viper envenomation can be misdiagnosed, and delayed recognition may delay appropriate management. Information about snake envenomation in Europe is mainly derived from studies in adults. A viper bite in children is rarely life-threatening, but it is particularly challenging due to the lower volume of distribution of venom in children compared to adults. Furthermore, symptoms could be heterogeneous and require close and careful monitoring for the onset of complications such as compartment syndrome. Although the venom of European vipers is considered mostly cytotoxic and hemotoxic, neurological symptoms are not so rare in children, especially affecting the cranial nerves.

A protocol for the management of viper bites in Europe was proposed, but a tailored and standardized protocol for children is still needed. It is important that management in children differs from that in adults, given the greater dangerousness of snake venom, the different clinical characteristics and therapeutic management.

Recently, efforts have been made to improve the pediatric approach to this issue. Several case reports in children were reported in literature, and a grading severity score for snakebitten children including the specific cutoff values of laboratory findings (e.g., white blood cells, neutrophil percentage) and specific management indications was created. Therefore, it is desirable that pediatricians use Audebert–Boels classification adapted to children by Marano et al. for the severity assessment and management of viper bites in children. Furthermore, larger studies are needed to validate the pediatric grading severity score and the VipGrade^®^ tool (https://www.toxicologie-clinique.org/vipgrade-tool/, accessed on 6 February 2025).

Furthermore, the differences in venomous snake species and available resources (including antivenoms) in each country highlight the need for a national snakebite register and standardized protocols for each European country to optimize the management of snakebites in children in Europe.

## Data Availability

Not applicable.

## Abbreviations

The following abbreviations are used in this manuscript:

WHO	World Health Organization
V.	Vipera
PLA2	Phospholipase A2
Ig	Immunoglobulin
LMWE	Low-molecular-weight heparin
